# “Has this been tested? Who has it helped? Who has it hurt?”: Public perceptions about California’s extreme risk protection order law

**DOI:** 10.1371/journal.pone.0334967

**Published:** 2025-11-04

**Authors:** Nicole Kravitz-Wirtz, Alexandra Dent, Shani Buggs, Amanda J. Aubel, Julia Lund, Garen Wintemute, Veronica A. Pear

**Affiliations:** Centers for Violence Prevention, Department of Emergency Medicine, School of Medicine, University of California Davis, Sacramento, California, United States of America; Uniformed Services University of the Health Sciences, UNITED STATES OF AMERICA

## Abstract

Extreme risk protection order (ERPO) laws in the United States temporarily suspend access to firearms by individuals judged to be at significant risk of harm to self or others. Evidence points toward preventive effects, but uptake of these laws remains lower than that which is likely needed to optimize their intended benefits on rates of firearm-related injury. To inform implementation efforts and policy refinements, we examined public awareness of and support for California’s ERPO law, barriers to its use, and possible alternative approaches. Using a general population sample of adults from the California Safety and Wellbeing Survey (*N* = 3531), with both closed-ended and open-ended questions, we provide updated prevalence estimates and narrative insights about the policy, with subgroup analyses by firearm ownership status and categories of race and ethnicity. Most respondents remained unaware of the law; however, after reading a brief policy description, majorities of the population, including firearm owners and respondents across all racial-ethnic subgroups, endorsed ERPOs as appropriate and said they would be willing to serve as petitioners for family members of concern. Barriers to use included knowledge gaps, particularly among non-firearm owners and Black, Latine, and Asian respondents; not trusting the system to be fair; and the perception that the scenarios of concern involved personal or family matters. More than one-third of respondents said the police were not a preferred means through which to initiate an order and a similar proportion favored holding onto a family member’s guns themselves instead of using an ERPO. Optimizing the lifesaving impacts of ERPOs will require addressing these layered concerns through coordinated and sustained investments in broader ecosystems of community safety while still facilitating firearm recovery where appropriate.

## Introduction

Extreme risk protection order (ERPO) laws in the United States (US) provide a civil court mechanism to temporarily suspend the possession and purchase of firearms and ammunition by individuals judged to be at significant risk of causing harm to themselves or others. ERPOs typically involve a two-stage process that includes: (1) an *ex-parte* order following judicial review of an emergency request, often lasting about two weeks to protect individuals from imminent harm until a hearing occurs, and (2) a final order issued by a judge after a full court hearing where all parties are notified and have an opportunity to be heard; these can last up to five years, depending on the jurisdiction. Barring other prohibitions, individuals who are subject to ERPOs have their firearm ownership rights fully restored upon expiration of the order. ERPO laws have so far been enacted in 21 states, the District of Columbia, and the US Virgin Islands. California enacted the first such law in 2016 (two states previously had more limited “risk-warrant” laws).

Nearly a decade later, use of ERPOs has increased, including in California where they are known as gun violence restraining orders (GVROs); e.g., California issued 2,762 orders in 2024, substantially more than the 85 orders issued during the law’s inaugural year and more than double the number issued in 2020 when the state legislature expanded the list of permissible petitioners from family or household members and law enforcement officers to other intimate partners, employers, coworkers, school employees, and teachers [1]. Evidence about the effectiveness of ERPOs is mixed but generally points toward preventive effects, especially decreased firearm suicide risk among individuals who are subject to ERPOs [2,3]. Yet ERPO uptake remains lower than that which is likely needed to optimize the policy’s intended benefits on rates of firearm death and injury [4].

Lack of awareness of these laws is often cited as a reason for their underutilization [5,6]. A national survey from 2019 found that most adults in gun-owning households reported being unsure whether their state had an ERPO law in force [6]. A 2020 survey in California likewise suggested that 2 in 3 adults had never heard of the state’s GVRO law, even when referred to more informally as a “red flag” law [5]. These and similar findings have led to calls for increased education and outreach efforts and, in California, were used as the basis for an 11 million dollar appropriation in the state’s Budget Act of 2021 for an 18-month public awareness campaign [7]. Recent research has assessed knowledge of ERPOs among selected permissible petitioners (e.g., law enforcement officers, medical and social service providers [8,9]); however, to our knowledge, general population estimates of ERPO awareness have yet to be updated.

ERPO utilization is likely further influenced by the extent to which potential petitioners perceive them as legitimate, responsive, and effective tools to ensure safety and health for a loved one who is in crisis or behaving in ways that may cause harm. These considerations may be especially pertinent for people with marginalized racial and other intersecting social identities who must contend with the reality that even civil procedures like ERPOs operate through the same criminal legal and immigration enforcement systems that disproportionately surveil, suspect, contact, arrest, and convict structurally vulnerable and minoritized people and communities [10–12]. We are aware of one study from California in which Black and Latine survey respondents were less likely than members of other racial and ethnic subgroups to endorse ERPO use, with suggestive evidence that mistrust at the interpersonal (e.g., law enforcement officers) and system (e.g., courts, legal system) levels were contributing factors [13]; however, additional investigations are needed to monitor the racial equity and behavioral health justice opportunities and impacts of ERPOs [14].

To expand extant literature, the current survey study presents updated prevalence estimates of the public’s knowledge of and support for using ERPOs as a tool to reduce firearm-related harms across multiple risk-based scenarios utilizing a general population sample of adults in California. Content analysis based on responses to open-ended questions also provide novel insights into barriers to the use of and possible alternatives to ERPOs. Particular emphasis is on subgroup differences by firearm ownership status and categories of race and ethnicity. As an early adopter of the law and a geographically and ethno-racially diverse state, California’s ongoing experience can be broadly informative for equitable implementation and refinement of ERPO laws and related firearm injury prevention policies and practices in other states and local jurisdictions throughout the country.

## Materials and methods

Data for this study come from the 2024 California Safety and Wellbeing Survey (CSaWS 2024), a repeated cross-sectional survey administered online in English and Spanish from November 4–25, 2024, by the survey research firm Ipsos. Survey respondents were members of the Ipsos KnowledgePanel, an online research panel widely used in health and safety research in the US. Recruitment to KnowledgePanel employs probability-based sampling methods with random-digit-dialing and address-based sampling from the US Postal Service’s Delivery Sequence File. KnowledgePanel members who are 18 years and older and non-institutionalized residents of California households were eligible to participate. Invitations were sent by e-mail; automatic reminders were e-mailed three days later and periodically thereafter. Of 5,841 invitations, 3,631 individuals completed the survey (completion rate, 62.2%); 100 cases were excluded from analysis for refusing more than half of the survey items (n = 13), providing an invalid or non-California zip code (n = 49), or refusing to provide a zip code (n = 38), resulting in a final sample of 3,531 respondents. The median survey completion time was 23 minutes.

The University of California, Davis Institutional Review Board approved all study procedures and issued a determination of exempt status. Given this determination, formal consent was not obtained; respondents read consent language at the beginning of the survey, including information about the study and study team, and progression to the next page of the online survey constituted consent. The study team did not have access to information that could directly identify individual respondents.

### Measures

Respondents were asked whether they had ever heard of a “gun violence restraining order (GVRO), extreme risk protection order (ERPO), or ‘red flag’ law.” Next, they read a short description of California’s GVRO law and were asked, “In general, do you think it would be appropriate for a judge to issue a GVRO” in five risk-based scenarios: when a person (1) is experiencing an emotional crisis, (2) has severe dementia or something like it, (3) has threatened to physically hurt themselves, (4) has threatened to physically hurt the survey respondent or someone else, and (5) has threatened to physically hurt a group of people. Response options were never, sometimes, usually, and always appropriate (dichotomized for analysis as never vs. at least sometimes) or don’t know. Those who answered “don’t know” in one or more scenarios were asked to write in what additional information they would need to help them decide. Respondents were then asked, “Would you personally be willing to ask a judge for a GVRO if a member of your family” was in each of the five scenarios. Response options were not at all, somewhat, and very willing (dichotomized for analysis as not at all vs. somewhat or very). Those who answered “not at all” in one or more scenarios were asked to select from a list of reasons why they were unwilling. To align with GVRO statute language, the prompts for the general appropriateness and personal willingness items included the qualifier, “Assume [the person/your family member] has or could get a gun and other options to protect against violence have failed or are not appropriate.” Respondents were also asked whether they would prefer to have the police ask a judge for a GVRO for them and whether they would prefer to hold onto their family member’s guns themselves instead of asking a judge for a GVRO.

Sociodemographic information for respondents is collected as part of initial KnowledgePanel membership and updated regularly. Race and ethnicity were jointly categorized using responses to two questions: “Are you Spanish, Hispanic, or Latino?” (hereafter, Latine) and then, “Please indicate what you consider your race to be” from one or more of the following US Census Bureau categories: White, Black or African American, American Indian or Alaska Native, Asian, and Native Hawaiian or Pacific Islander. Respondents who did not endorse Spanish, Hispanic, or Latino ethnicity and who identified as American Indian or Alaska Native (n = 10), Native Hawaiian or other Pacific Islander (n = 5), or more than one race (n = 86) were combined into an “other/multi-race” category to maintain sufficient sample size for analysis. Other sociodemographic characteristics include age, sex, educational attainment, and household income. In addition, respondents were asked a pair of questions about whether they or anyone they live with currently owns a gun and categorized accordingly as firearm owners, non-owners who live with firearm owners, and non-owners in homes without guns. Full-text survey items are in S1 Appendix.

### Analysis

We calculated weighted percentages and 95% confidence intervals (CI) for each closed-ended survey item or cross-tabulation of items using a post-stratification statistical weight variable provided by Ipsos. This weight variable adjusts for the initial probability of selection into KnowledgePanel and for survey non-response and over- or under-coverage using post-stratification raking ratio adjustments. With weighting, our estimates are designed to be statistically representative of the adult population of California, as reflected in the 2018–2022 American Community Survey. Comparisons by categories of race and ethnicity and firearm ownership status were conducted using two-sample t-tests (p < 0.05) with the weighted population. Statistical analyses were conducted in Stata statistical software version 18.5 (StataCorp). Open-ended survey items with write-in responses were abstracted, translated from Spanish into English through a web-based translation engine, as appropriate, and thematically coded by two authors (AD, NKW) in Microsoft Excel version 2501.

## Results

Of 3,531 respondents (mean [SD] age, 48.5 [17.3] years), 48.2% (95% CI, 45.7–50.7) were men and 39.7% (95% CI, 37.3–42.1) identified as White, 35.9% (95% CI, 33.5–38.4) as Latine, 15.4% (95% CI, 13.5–17.5) as Asian, 5.2% (95% CI, 4.2–6.5) as Black, and 3.8% (95% CI, 2.9–5.0) as other/multi-race. Firearm owners were 15.4% (95% CI, 13.8–17.2) of the sample; an additional 11.3% (95% CI, 9.6–13.1) did not personally own a gun but lived with someone who did. Sociodemographic characteristics of respondents are in S1 Table.

### Awareness

Approximately two-thirds (67.4%; 95% CI, 65.1–69.7) of respondents reported never having heard of a GVRO, ERPO, or “red flag” law (**Table 1**). Non-owners in homes without guns (71.0%; 95% CI, 69.3–73.6) and non-owners who live with firearm owners (69.8%; 95% CI, 62.0–76.6) were more likely to be unaware of the law compared with firearm owners (50.1%; 95% CI, 44.2–56.0). Across categories of race and ethnicity, the percentage of respondents who had never heard of the law was highest among Black respondents (77.1%; 95% CI, 66.4–85.2), followed by respondents who identified as Asian (75.3%; 95% CI, 68.4–81.2), Latine (73.2%; 95% CI, 69.3–76.8), other/multi-race (65.3%; 95% CI, 51.3–77.1), and White (58.0%; 95% CI, 54.5–61.4).

**Table 1:** Public Awareness of GVROs Among California Adults, by Firearm Ownership Status, California Safety and Wellbeing Survey, 2024 (n = 3,531).

Total	Aware of the GVRO law			Unaware of the GVRO law		
	Unweighted *n*	Weighted % (95% CI)	*P* value	Unweighted *n*	Weighted % (95% CI)	*P* value
	1,283	32.0 (29.8-34.4)		2,240	67.4 (65.1-69.7)	
Firearm ownership status						
Non-owners in homes without guns	800	28.5 (25.9-31.2)	<0.001^2^	1,594	71.0 (68.3-73.6)	<0.001^2^
Firearm owners	326	49.0 (43.1-54.9)	<0.001^1^ <0.001^3^	290	50.1 (44.2-56.0)	<0.001^1^ <0.001^3^
Non-owners who live with firearm owners	104	30.2 (23.5-38.0)	<0.001^2^	229	69.8 (62.0-76.6)	<0.001^2^
Categories of race and ethnicity						
White	782	41.2 (37.8-44.7)		998	58.0 (54.5-61.4)	
Black	53	21.0 (13.4-31.4)		161	77.1 (66.4-85.2)	
Latine	331	26.4 (22.9-30.3)		784	73.2 (69.3-76.8)	
Asian	76	24.6 (18.8-31.5)		237	75.3 (68.4-81.2)	
Other/Multi	41	34.7 (22.9-48.8)		60	65.3 (51.3-77.1)	

Note: Percentages may not total to 100% because refusals and don’t know responses are not shown

^(1)^Significant difference from non-owners in homes without guns

^(2)^Significant difference from firearm owners

^(3)^Significant difference from non-owners who live with firearm owners

### General appropriateness

Across all five risk-based scenarios, most respondents reported that, in general, it was sometimes, usually, or always appropriate for a judge to issue a GVRO, with levels of endorsement ranging from 74.6% (95% CI, 72.2–76.9), including 55.7% (95% CI, 53.2–58.3) of respondents who said it was usually or always appropriate, in cases where the person has severe dementia to 82.0% (95% CI, 79.9–84.0), including 73.2% (95% CI, 70.8–75.4) of respondents who said it was usually or always appropriate, when the person has threatened to harm someone else (**Table 2** and S2 Table). The percentage of respondents reporting that a GVRO was at least sometimes appropriate was highest among firearm owners for all risk-based scenarios (depending on the scenario, 80.6%−89.9%), while support was slightly lower among non-owners in homes without guns (depending on the scenario, 73.0%−81.0%; **Table 3**); though, in four of five scenarios, the percentage of respondents who said a GVRO was usually or always appropriate was highest among non-owners who live with firearm owners (depending on the scenario, 58.2%−83.1%; S3 Table). Respondents who identified as Black or Latine were mostly supportive but relatively less so compared with other racial and ethnic subgroups: depending on the scenario, 61.4%−69.7% of Black respondents and 65.9%−74.6% of Latine respondents said that, in general, a GVRO was sometimes, usually, or always appropriate compared with 75.5%−91.7% endorsement across other racial and ethnic subgroups (**Table 4**); in four of five scenarios, the percentage reporting that a GVRO was usually or always appropriate was also relatively lower among Black and Latine respondents (depending on the scenario, 43.5%−62.1% Black respondents and 48.5%−63.4% Latine respondents; S4 Table).

**Table 2:** Perceived Appropriateness of GVROs, In General, by Risk-Based Scenario, California Safety and Wellbeing Survey, 2024 (n = 3,531).

	Never appropriate		Sometimes/usually/always appropriate	
	Unweighted *n*	Weighted % (95% CI)	Unweighted *n*	Weighted % (95% CI)
Person is experiencing an emotional crisis	395	13.8 (12.0-15.8)	2,847	75.3 (72.9-77.6)
Person has severe dementia or something like it	429	13.8 (12-15.7)	2,803	74.6 (72.2-76.9)
Person threatened to physically hurt themselves	361	11.8 (10.2-13.6)	2,937	79.4 (77.1-81.4)
Person threatened to physically hurt someone else	318	10.2 (8.7-12.0)	3,016	82.0 (79.9-84.0)
Person threatened to physically hurt a group of people	332	11.1 (9.5-12.9)	2,997	80.9 (78.6-82.9)

Note: Percentages may not total to 100% because refusals and don’t know responses are not shown

**Table 3:** Perceived Appropriateness of GVROs, In General, by Risk-Based Scenario and Firearm Ownership Status, California Safety and Wellbeing Survey, 2024 (n = 3,531).

Firearm ownership status	Never appropriate		Sometimes/usually/always appropriate		
	Unweighted *n*	Weighted % (95% CI)	Unweighted *n*	Weighted % (95% CI)	*Ρ* value
Person is experiencing an emotional crisis					
Non-owners in homes without guns	263	13.2 (11.1-15.7)	1,932	75.7 (72.8-78.4)	
Firearm owners	62	12.3 (8.4-17.6)	521	80.6 (74.5-85.5)	
Non-owners who live with firearm owners	36	14.4 (9.3-21.6)	285	79.7 (71.5-85.9)	
Person has severe dementia or something like it					
Non-owners in homes without guns	314	15.1 (12.9-17.6)	1,873	73.0 (70.0-75.8)	<.001^2^
Firearm owners	50	8.7 (5.6-13.4)	537	85.3 (79.8-89.6)	<.001^1^
Non-owners who live with firearm owners	39	13.6 (8.6-20.8)	278	78.1 (69.9-84.5)	
Person threatened to physically hurt themselves					
Non-owners in homes without guns	262	12.5 (6.7-21.9)	1,969	78.2 (75.4-80.7)	0.004^2^ 0.03^3^
Firearm owners	46	10.1 (6.5-15.6)	547	86.0 (80.6-90.1)	0.004^1^
Non-owners who live with firearm owners	28	11.1 (6.6-18.2)	298	85.8 (78.2-91.0)	0.03^1^
Person threatened to physically hurt someone else					
Non-owners in homes without guns	231	11.0 (9.1-13.1)	2,023	81.0 (78.4-83.4)	0.002^2^
Firearm owners	40	8.8 (5.4-14.	564	89.2 (83.9-92.9)	0.002^1^
Non-owners who live with firearm owners	22	7.4 (4.0-13.2)	301	86.9 (79.6-91.8)	
Person threatened to physically hurt a group of people					
Non-owners in homes without guns	245	12.3 (10.3-14.6)	2,007	79.4 (76.6-81.9)	<.001^1^ 0.03^3^
Firearm owners	41	8.1 (4.9-13.2)	561	89.9 (84.8-93.3)	<.001^1^
Non-owners who live with firearm owners	19	6.2 (3.2-11.9)	303	87.0 (79.5-92.0)	0.03^1^

Note: Percentages may not total to 100% because refusals and don’t know responses are not shown

^(1)^Significant difference from non-owners in homes without guns

^(2)^Significant difference from firearm owners

^(3)^Significant difference from non-owners who live with firearm owners

**Table 4:** Perceived Appropriateness of GVROs, In General, by Risk-Based Scenario and Categories of Race and Ethnicity, California Safety and Wellbeing Survey, 2024 (n = 3,531).

Categories of race and ethnicity	Never appropriate		Sometimes/usually/always appropriate	
	Unweighted *n*	Weighted % (95% CI)	Unweighted *n*	Weighted % (95% CI)
Person is experiencing an emotional crisis				
White	122	8.7 (6.7-11.2)	1,560	83.7 (80.5-86.5)
Black	40	20.7 (12.9-31.4)	150	67.0 (55.5-76.8)
Latine	187	19.5 (16.0-23.4)	74	74.6 (55.7-87.3)
Asian	36	12.0 (7.6-18.4)	254	79.3 (72.1-84.9)
Other/Multi	10	11.6 (4.9-24.9)	84	76.0 (59.7-87.2)
Person has severe dementia or something like it				
White	127	8.4 (6.6-10.7)	1,553	83.5 (80.3-86.2)
Black	43	20.0 (12.5-30.3)	142	61.4 (49.8-71.8)
Latine	203	18.4 (15.2-22.1)	779	65.9 (61.6-70.0)
Asian	43	15.3 (10.2-22.2)	248	75.7 (68.3-81.8)
Other/Multi	13	10.6 (4.3-23.8)	81	78.0 (61.5-88.7)
Person threatened to physically hurt themselves				
White	86	5.8 (4.3-7.8)	1,623	89.0 (86.3-91.2)
Black	40	24.4 (15.4-36.6)	155	67.5 (55.6-77.5)
Latine	193	17.4 (14.3-20.9)	807	68.1 (63.9-72.1)
Asian	34	10.4 (6.5-16.3)	264	83.3 (76.6-88.4)
Other/Multi	8	9.1 (3.2-23.2)	88	85.2 (69.6-93.6)
Person threatened to physically hurt someone else				
White	65	4.2 (3.0-5.8)	1,659	91.3 (88.8-93.2)
Black	38	22.0 (13.3-34.1)	161	69.7 (57.7-79.6)
Latine	172	15.5 (12.6-18.9)	842	71.9 (67.7-75.7)
Asian	32	9.3 (5.6-15.2)	268	85.7 (79.3-90.4)
Other/Multi	11	10.8 (4.3-24.5)	86	83.8 (68.4-92.5)
Person threatened to physically hurt a group of people				
White	70	4.0 (2.9-5.5)	1,659	91.7 (89.3-93.6)
Black	42	25.9 (16.4-38.3)	157	65.6 (53.4-76.0)
Latine	177	17.7 (14.5-21.3)	830	69.2 (64.9-73.2)
Asian	32	9.1 (5.4-14.8)	266	84.7 (78.2-89.5)
Other/Multi	11	10.7 (4.2-24.4)	85	83.6 (68.3-92.4)

Note: Percentages may not total to 100% because refusals and don’t know responses are not shown

Among the 15.5% (95% CI: 13.6–17.6) of respondents who said they “didn’t know” when asked whether a GVRO was appropriate in at least one scenario, approximately half (N = 243) provided write-in responses about what they needed to help them decide. Respondents most often noted a need for additional information about GVROs in general, particularly among Spanish language respondents, and about the characteristics and conditions of the individuals and scenarios in question more specifically; mental health status and engagement with medical or mental health services, including as possible alternatives to GVROs, were especially common: *“I would think making arrangements for an appointment to see a therapist and fully discuss what is [g]oing on with that particular individual’s life or situation.”* The need for more information was likewise prevalent in relation to dementia and firearm-related harm: *“I need more reasons as to why dementia would be a factor to remove gun ownership.”* The write-in responses also suggested a need for additional clarification about due process rights given recurrent concerns related to *“2nd [A]mendment infringement”* and GVROs being *“misused to take someone[’s] constitutional rights away.”*

While less common, other topics to aid decision making were also noted. These included the need to address perceptions that the risk-based scenarios involved private or family matters (e.g., *“Not my place to decide”*), concerns about law enforcement (e.g., *“…I have seen in the community that when something happens, in the examples that you mention, all the time the local police, the [S]heriff’s department, the CHP...respond to situations like these with excessive aggression…”* [translated from Spanish]), and questions about existing evidence of effectiveness (e.g., *“Has this been tested? Who has it helped? Who has it hurt?”*). A summary of primary and secondary themes with illustrative quotes is in **Table 5**.

**Table 5:** Primary and Secondary Themes and Illustrative Quotes for What Would Aid Decision-making About GVRO Appropriateness, California Safety and Wellbeing Survey, 2024.

Primary Themes	Secondary Themes	Illustrative Quotes
**Knowledge and awareness**	Policy and process	“I don’t really know about GVRO[s] so I can’t say what is right or wrong.”
		“I just don’t know enough about it.”
		“Necesitaría saber más sobre esto de GVRO.” [I would need to know more about this GVRO thing.]
	Evidence of effectiveness	“Has this been tested? Who has it helped? Who has it hurt?”
		“More information or statistics from similar occurrences.”
	Characteristics of individuals of concern	“The mental and health history of the individual.”
		“I’d need to know if the person had prior tendencies for violence or are in danger of self-harm.”
		“Background info on person’s behaviors and or legal problems. Legal grounds for bringing the community actions. What follow-up will be ordered, by whom, to what end?”
	Conditions related to risk-based scenarios	“I would think making arrangements for an appointment to see a therapist and fully discuss what is doing on with that particular individual’s life or situation.”
		“Circumstances always play a key role”
**Personal or family matter**		“Not my place to decide.”
		“I believe a person has the right to terminate their life if they wish to after appropriate counseling.”
**Constitutionality**	Second Amendment	“Does it violate the 2nd Amendment?”
		“I don’t feel like California needs any more power to take guns away from law abiding citizens.”
	Due process	“More info on red flag laws...you can just submit a petition to court, w[ith] no real accusation of a crime to take someone’s property? Don’t like it.”
		“I worry about the civil rights of the individual and would have to know more facts before I would side with this law. I personally don’t agree with this law as I am worried that it can be used as a weapon or threat to anyone.”
		“Evidence that the person threatened to hurt. If it is a disagreement between 2 people I believe there needs to be more supporting evidence of a threat like other witnesses or texts. When people dislike each they can lie or exaggerate to punish the other person.”
**Risk for firearm-related harm**	Dementia	“Dementia has nothing to do with a judge ordering a GVRO so to me that seems not needed.”
		“I need more reasons as to why dementia would be a factor to remove gun ownership.”
		“I’m not familiar with dementia or how it manifests, so can’t judge how likely someone with it will be likely to hurt themselves or others.”
	Emotional crisis	“‘Emotional crisis’ is a phrase people throw around too easily. If the person admits they’re having an emotional crisis, that’s one thing. Onlookers? No.”
**System-level trust or mistrust**	Law enforcement	“En mis experiencias viviendo en california, he visto en la comunidad, que cuando algo pasa, en los ejemplos que ustedes mencionan, todo el tiempo la policia local el departamento del sheriff, el chp. Todo el timpo responden a situaciones como estas con un exesso de agrecividad, por lo tanto en eso baso mis respuestas, tal vez no sean las que ustedes buscan.” [In my experiences living in California, I have seen in the community that when something happens, in the examples that you mention, all the time the local police, the [S]heriff’s department, the CHP. All the time they respond to situations like these with excessive aggression, so that’s what I base my responses on, maybe they are not what you are looking for.]
		“I’m not familiar with some of the programs, but I would think that the police would know the correct persons to contact.”

Note: If a respondent said they “don’t know” whether a GVRO is appropriate in at least one risk-based scenario, they were asked, “In a few words or sentences, what additional information would you need to help you decide?”

### Personal willingness

When asked to consider a family member of concern more specifically, more than three in four respondents, across all five risk-based scenarios, said they would be somewhat or very willing to personally ask a judge for a GVRO (depending on the scenario, 76.8%−86.7%; S5 and S6 Tables). Subgroup differences by firearm ownership status and categories of race and ethnicity were less pronounced than those for the general appropriateness of GVROs, and no uniform subgroup-specific patterns in personal willingness were observed across scenarios. Nearly one-third (28.9%; 95% CI: 26.6–31.4) of respondents reported that they were not at all willing to ask a judge for a GVRO for a family member in at least one scenario. Respondents who identified as Latine or other/multi race more often said they were not at all willing to personally petition for a GVRO across all five scenarios (depending on the subgroup, 11.5%−12.1%) compared with other racial and ethnic subgroups (depending on the 5.7%−9.4%). Levels of unwillingness to personally petition for a GVRO did not meaningfully differ by firearm ownership status.

### Barriers to use and possible alternatives

Among respondents who reported being not at all willing to ask a judge for a GVRO in at least one scenario, nearly half (49.1%; 95% CI, 44.0–54.1) said it was because they did not know enough about GVROs. This percentage was larger among non-owners in homes without guns (57.8%; 95% CI, 51.6–63.7; **Table 6**) and respondents who identified as Black (52.2%; 95% CI, 32.4–71.2), Latine (57.8%; 95% CI, 49.9–65.2), or Asian (62.2%; 95% CI, 46.8–75.5; **Table 7**). Not trusting the system to be fair was the most common reason for being not at all willing to use a GVRO among firearm owners (49.1%; 95% CI, 37.3–61.0) and the second most frequently endorsed reason overall (23.6%; 95% CI, 19.7–28.1) and among respondents who identified as Asian, Black, and White (depending on the subgroup, 28.0%−35.1%). The perception that the scenarios involved personal or family matters was also endorsed as a reason for being not at willing to use a GVRO by nearly one-quarter (23.4%; 95% CI, 19.8–28.5) of all respondents and approximately half of both firearm owners (47.9%; 95% CI, 36.0–60.0) and respondents who identified as other/multi race (49.1%; 95% CI, 26.9–71.6).

**Table 6:** Reasons Not At All Willing to Ask a Judge for a GVRO for a Family Member, by Firearm Ownership Status, California Safety and Wellbeing Survey, 2024 (n = 3,531).

	Total (n = 902)		Non-owners in homes without guns (n = 576)			Firearm owners (n = 170)			Non-owners who live with firearm owners (n = 85)		
	Unweighted *n*	Weighted % (95% CI)	Unweighted *n*	Weighted % (95% CI)	*P* value	Unweighted *n*	Weighted % (95% CI)	*P* value	Unweighted *n*	Weighted % (95% CI)	*P* value
Don’t know enough about GVROs	404	49.1 (44.0-54.1)	300	57.8 (51.6-63.7)	<.001^2^	36	13.8 (8.7-21.2)	<.001^1^ <.001^3^	36	49.4 (33.6-65.3)	<.0001^2^
These are personal/family matters	119	13.4 (10.3-17.3)	86	16.4 (23.2-21.7)	.001^2^	15	5.8 (2.8-11.7)	.001^1^	7	8.4 (2.6-24.0)	
Don’t trust the system will be fair	221	19.4 (16.1-23.3)	107	14.1 (10.8-18.2)	<.001^2^	73	42.7 (31.4-54.8)	<.001^1^ <.001^3^	24	14.9 (8.3-25.4)	<.0001^2^
Worried about due process rights	168	19.2 (15.6-23.4)	101	16.2 (12.3-21.1)	.013^2^	39	32.6 (21.8-45.7)	.013^1^	18	24.2 (13.0-40.4)	
Don’t want to involve the court	228	23.6 (19.7-28.1)	114	18.1 (13.9-23.4)	<.001^2^	76	49.1 (37.3-61.0)	<.001^1^ <.001^3^	19	18.1 (8.1-35.5)	<.0001^2^
Never appropriate to take someone’s guns	226	23.4 (19.8-28.5)	123	17.0 (12.9-39.3)	<.001^2^	64	47.9 (36.0-60.0)	.001^1^	26	31.0 (18.3-47.5)	
Worried about retaliation	88	12.0 (9.0-15.8)	32	5.6 (3.5-8.8)	<.001^2^	36	31.6 (20.9-44.8)	<.001^1^ .017^3^	8	12.4 (5.2-26.6)	.017^2^
Some other reason	98	7.5 (5.5-10.3)	60	7.5 (5.0-11.0)		23	9.2 (3.1-24.3)		8	9.2 (4.6-17.5)	

Note: Percentages of respondents who did not endorse each reason and refusals are not shown

^(1)^Significant difference from non-owners in homes without guns

^(2)^Significant difference from firearm owners

**Table 7:** Reasons Not At All Willing to Ask a Judge for a GVRO for a Family Member, by Categories of Race and Ethnicity, California Safety and Wellbeing Survey, 2024 (n = 3,531).

	Total (n = 902)		White (n = 382)		Black (n = 66)		Latine (n = 337)		Asian (n = 80)		Other/Multi (n = 37)	
	Un-weighted *n*	Weighted % (95% CI)	Un-weighted *n*	Weighted % (95% CI)	Un-weighted *n*	Weighted % (95% CI)	Un-weighted *n*	Weighted % (95% CI)	Un-weighted *n*	Weighted % (95% CI)	Un-weighted *n*	Weighted % (95% CI)
Don’t know enough about GVROs	404	49.1 (44.0-54.1)	132	35.2 (28.0-43.2)	35	52.2 (32.4-71.2)	182	57.8 (49.9-65.2)	43	62.2 (46.8-75.5)	12	27.6 (12.6-50.1)
These are personal/family matters	226	23.4 (19.8-28.5)	119	30.0 (23.4-37.5)	13	24.7 (10.2-48.7)	59	15.7 (10.9-22.1)	23	23.8 (13.0-39.5)	12	49.1 (26.9-71.6)
Don’t trust the system will be fair	228	23.6 (19.7-28.1)	117	35.1 (27.8-43.1)	17	28.0 (13.8-48.6)	60	11.5 (7.9-16.4)	24	29.0 (17.1-44.8)	10	25.7 (11.3-48.5)
Worried about due process rights	221	19.4 (16.1-23.3)	124	30.4 (23.9-37.7)	11	10.9 (4.5-24.2)	60	12.7 (8.7-18.2)	17	18.3 (9.5-32.5)	9	16.1 (7.0-32.8)
Don’t want to involve the court	168	19.2 (15.6-23.4)	83	23.6 (17.7-30.8)	8	8.9 (3.6-20.4)	55	14.5 (10.1-20.3)	15	20.0 (10.2-35.4)	7	37.4 (17.1-63.5)
Never appropriate to take someone’s guns	88	12.0 (9.0-15.8)	49	18.7 (13.1-26.1)	4	11.4 (3.5-31.5)	26	7.5 (4.3-12.9)	5	8.2 (2.5-23.4)	4	15.2 (3.6-46.6)
Worried about retaliation	119	13.4 (10.3-17.3)	34	11.3 (6.7-18.5)	6	5.2 (1.9-13.5)	56	13.9 (9.7-19.5)	16	21.1 (10.8-37.3)	7	11.7 (4.6-26.5)
Some other reason	98	7.5 (5.5-10.3)	52	11.0 (1.3-16.1)	6	7.8 (2.0-11.1)	28	5.8 (3.3-10.0)	6	4.7 (1.3-16.1)	6	10.7 (3.9-26.3)

Note: Percentages of respondents who did not endorse each reason and refusals are not shown

Most (59.9%; 95% CI, 57.4–62.4) respondents said they would prefer to have the police petition for a GVRO for them, but support was not uniformly high (**Table 8**). Approximately half of both firearm owners (46.7%; 95% CI, 40.8–52.7) and respondents who identified as Black (50.5%; 95% CI, 39.5–61.6) did not favor having the police petition for an order on their behalf. Additionally, an estimated one in three (35.5%; 95% CI, 33.1–37.9) respondents overall and most firearm owners (69.0%; 95% CI, 63.3–74.1) said they would prefer to hold onto their family member’s guns themselves instead of asking a judge for a GVRO (**Table 9**). Holding onto a family member’s guns was also endorsed relatively more frequently among respondents who identified as Black (40.7%; 95% CI, 30.1–52.3) or other/multi-race (45.5%; 95% CI, 31.8–59.6).

**Table 8:** Preference for Police Petitioning for a GVRO, by Firearm Ownership Status and Categories of Race and Ethnicity, California Safety and Wellbeing Survey, 2024 (n = 3,531).

Total	Yes, prefer to have police petition			No, do not prefer to have police petition		
	Unweighted *n*	Weighted % (95% CI)	*P* value	Unweighted *n*	Weighted % (95% CI)	*P* value
	2,188	59.9 (57.4-62.4)		1,276	37.8 (35.4-40.3)	
Firearm Ownership Status						
Non-owners in homes without guns	1,527	62.3 (59.3-62.3)	.003^2^	821	34.9 (32.0-37.9)	<.001^2^
Firearm owners	357	52.1 (46.1-68.0)	.003^1^/.036^3^	253	46.7 (40.8-52.7)	<.001^1^/ < 0.01^3^
Non-owners who live with firearm owners	213	62.3 (54.4-70.5)	.036^2^	119	37.0 (29.3-45.4)	
Race and Ethnicity						
White	1,116	59.0 (55.4-62.5)		630	37.9 (34.5-41.5)	
Black	96	47.1 (36.2-58.3)		113	50.5 (39.5-61.6)	
Latine	718	62.3 (58.9-67.4)		386	35.5 (31.4-39.8)	
Asian	191	58.1 (50.6-65.2)		115	38.6 (31.6-46.1)	
Other/Multi	67	62.3 (47.0-75.5)		32	37.3 (24.1-52.6)	

Note: Percentages may not total to 100% because refusals and don’t know responses are not shown

(1) Significant difference from non-owners in homes without guns

(2) Significant difference from firearm owners

(3) Significant difference from non-owners who live with firearm owners

**Table 9:** Preference for Holding onto a Family Member’s Gun(s), by Firearm Ownership Status and Categories of Race and Ethnicity, California Safety and Wellbeing Survey, 2024 (n = 3,531).

Total	Yes, prefer to hold onto guns			No, do not prefer to hold onto guns		
	Unweighted *n*	Weighted % (95% CI)	*P* value	Unweighted *n*	Weighted % (95% CI)	*P* value
	1,203	35.5 (33.1-37.9)		2,225	62.1 (59.6-64.5)	
Firearm Ownership Status						
Non-owners in homes without guns	566	25.4 (22.8-28.2)	<.001^2^/ < .001^3^	1,779	71.8 (68.9-74.6)	<.001^2^/ < .001^3^
Firearm owners	418	69.0 (63.3-74.1)	<.001^2^/ < .001^3^	195	30.3 (25.1-35.9)	<.001^1^/ < .001^3^
Non-owners who live with firearm owners	141	45.0 (37.0-53.3)	<.001^2^/ < .001^3^	188	53.3 (45.0-61.4)	<.001^2^/ < .001^3^
Race and Ethnicity						
White	619	38.0 (64.6-41.6)		1,133	60.1 (56.6-63.6)	
Black	75	40.7 (30.1-52.3)		132	56.6 (45.2-67.4)	
Latine	377	33.3 (29.3-37.5)		715	63.6 (59.2-67.7)	
Asian	88	29.8 (23.4-67.2)		221	68.4 (60.9-75.0)	
Other/Multi	44	45.5 (31.8-59.6)		54	51.1 (37.0-65.0)	

Note: Percentages may not total to 100% because refusals and don’t know responses are not shown

(1) Significant difference from non-owners in homes without guns

(2) Significant difference from firearm owners

(3) Significant difference from non-owners who live with firearm owners

## Discussion

Many firearm injury prevention strategies, including ERPOs, rely in part on preventive actions initiated by health, safety, and other care providers who come into contact with individuals at elevated risk of causing harm to self or others. However, for a variety of individual but also institutional and societal reasons, many individuals of concern do not come to the attention of frontline providers or, if they do, may be reluctant to voluntarily relinquish firearm access. For these individuals, it is the broader public and particularly people with whom they have frequent interactions (e.g., family, household members, coworkers) who may be the first to recognize—and serve as credible sources for communicating—that they are in crisis or behaving dangerously. A prior survey in California, for example, estimated that one in five people know at least one person, usually a friend or extended family member, whom they perceived to be at risk of violence to self or others; among respondents living with the person of concern, one-quarter reported household firearm ownership [15].

Yet, public awareness of California’s nearly decade-old ERPO law remains low and effectively unchanged since it was last assessed. Similar to statewide estimates from 2020 [5], approximately two in three (67.4%) respondents reported never having heard of the policy. This lack of knowledge about the law was further underscored by respondents who said they were not at all willing to use an ERPO in one or more risk-based scenarios (28.9%), half of whom said it was because they did not know enough about the law. Relatively larger shares of those who did not own firearms and respondents who identified as Asian, Black, or Latine cited lack of knowledge as a reason for their unwillingness to use an ERPO, indicating opportunities for tailored and culturally responsive outreach efforts to these populations. Write-in survey responses also suggested that gaps in knowledge about the law, especially among Spanish language survey respondents, and a need for additional information about the characteristics and conditions of the individuals and scenarios of concern (e.g., mental or emotional health status and service use) were common barriers to decision making about ERPO use.

Notwithstanding gaps in awareness and knowledge, after reading a brief description of the law, sizable majorities of the population, including firearm owners and non-owners as well as respondents across all racial and ethnic subgroups, endorsed ERPOs as an appropriate violence prevention tool in general (depending on the subgroup and scenario, 61.4%−91.7%). Such widespread support aligns with prior statewide survey estimates [5], as well as national research indicating that many firearm injury prevention policies, including temporary firearm removal policies, have broad public support and that general consensus exists between firearm owners and non-owners; though, consistent with this study, national survey respondents who identified as Black, while still mostly supportive, were relatively less so when such policies were initiated by law enforcement [16,17]. More significantly, perhaps, majorities of all subgroups of respondents in this study reported being personally willing to use an ERPO for a family member across an array of risk-based scenarios that included a loved one experiencing cognitive decline, emotional distress, and threats to self and others (depending on the subgroup and scenario, 65.4%−91.1%); though, relatively lower levels of support for the dementia scenario coupled with the write-in responses suggest a need to raise awareness about the risks of firearm-related harm in the context of dementia, consistent with past research [18].

Widespread policy support bodes well for the positive impacts of increased ERPO public awareness and educational campaigns; though, the effectiveness of such existing efforts remains unclear. Translating gains in knowledge into actual ERPO use for real people at times of high risk is more likely if outreach efforts are responsive to the values and experiences of communities most affected by firearm violence and suicide. This requires prioritizing research involving significant and sustained community and careholder engagement, including people with elevated risk for and direct experience of violence and injury, so that ERPO implementation and related firearm injury prevention strategies are designed and delivered to truly address community needs. It also means directly confronting the systems, forces, and narratives that fuel diverse ideological and experiential concerns related to procedural justice. Nearly one-quarter of respondents said they would not use an ERPO because they did not trust the system to be fair. For some, distrust may stem from generalized concerns about privacy and the Second Amendment; for others, it may reflect lived experience of systemic inequities, including racialized injustice, police violence, and health and criminal legal system failures [13]. Addressing these layered concerns requires attention to their distinct roots, as well as leveraging opportunities to engage coordinated non-criminal legal system-led strategies for responding to and supporting individuals at risk of harm while still facilitating ERPO service and firearm recovery where appropriate.

These expanded strategies build on the proactive, non-punitive spirit of ERPO laws. Examples of these approaches might include community-based workers, such as unarmed crisis (co-)responders, violence interrupters, legal system navigators, and court advocates, who might serve as culturally responsive liaisons to information about ERPOs and other supportive resources and responses for individuals of concern and their families, as well as structured options for temporary, voluntary firearm storage with family members or other trusted sources such as federally licensed firearm ranges/retailers [19]. This study did not examine the feasibility of these broader strategies, but they are critical areas for further investigation considering that more than one in three respondents said the police were not a preferred means through which to initiate an order and that a similar share of respondents favored holding onto a family member’s guns themselves instead of petitioning for an ERPO. It will be critical, however, to not simply transfer responsibility—and liability [19,20]—for the societal good of safety from the collective to the individual. Adequately resourcing the social and health systems through which such expanded services are provided and investing in a coordinated and sustainable ecosystem of public safety rooted in compassion, care, and support remains essential for equitably and sustainably preventing and reducing firearm-related harms.

### Limitations

This study has limitations. First, self-reported data is subject to nonresponse and social desirability biases; however, item nonresponse in this study was low overall, and research suggests that completion rates are higher and social desirability bias is lower in online panel surveys than in random-digit-dial telephone surveys [21]. Second, although we think these findings can inform understandings of ERPO implementation and refinement in other states, variations in firearm ownership, the cultural acceptability of firearms, firearm violence, and firearm regulations should be considered when attempting to generalize these results. Third, collapsing response categories (somewhat, usually, and always appropriate; somewhat and very willing) may mask more nuanced differences in the extent of support for ERPOs. However, we posit that any level of endorsement is not only noteworthy but may also be valuable for changing behavior via perceived social norms. Finally, although we measured self-reported support for use, the extent to which this translates into real-world behavior is unclear and will require community responsive messaging and implementation options.

## Conclusion

Findings from this statewide general population survey study contribute to a growing evidence base supporting the broad public acceptability of and willingness to use ERPOs to prevent firearm-related harms. While promising, self-reported barriers to use and possible alternative approaches indicate that ERPOs are not a panacea. Optimizing the lifesaving impacts of ERPOs and related firearm injury prevention strategies will require ongoing attention to and sustained investment in coordinated, multi-sector, equity conscious, and community responsive public health and safety approaches.

## Supporting information

S1 Appendix Detailed question wording and response options, California Safety and Wellbeing Survey (CSaWS), 2024. (PDF)

S1 Table Sociodemographic Characteristics of Respondents, by Firearm Ownership Status, California Safety and Wellbeing Survey, 2024 (n = 3,531). (PDF)

S2 Table Perceived Appropriateness of GVROs, In General, by Risk-Based Scenario, California Safety and Wellbeing Survey, 2024 (n = 3,531). (PDF)

S3 Table Perceived Appropriateness of GVROs, In General, by Risk-Based Scenario and Firearm Ownership Status, California Safety and Wellbeing Survey, 2024 (n = 3,531). (PDF)

S4 Table Perceived Appropriateness of GVROs, In General, by Risk-Based Scenario and Categories of Race and Ethnicity, California Safety and Wellbeing Survey, 2024 (n = 3,531). (PDF)

S5 Table Perceived Willingness to Personally Petition for a GVRO for a Family Member, by Risk Scenario, Firearm Ownership Status (Panel A) and Categories of Race and Ethnicity (Panel B), California Safety and Wellbeing Survey, 2024 (n = 3,531). (PDF)

S6 Table Perceived Willingness to Personally Petition for a GVRO for a Family Member, by Risk Scenario, Firearm Ownership Status (Panel A) and Categories of Race and Ethnicity (Panel B), California Safety and Wellbeing Survey, 2024 (n = 3,531). (PDF)
