# Supplementary material for: “Has this been tested? Who has it helped? Who has it hurt?”: Public perceptions about California’s extreme risk protection order law
Source: PLoS One. 2025 Nov 4;20(11):e0334967. doi: 10.1371/journal.pone.0334967 (PMC12585041; doi:10.1371/journal.pone.0334967)
Supplement: S1 Appendix — (PDF) [file pone.0334967.s001.pdf]

# “Has this been tested? Who has it helped? Who has it hurt?”: Public perceptions about California’s Extreme Risk Protection Order law

Nicole Kravitz-Wirtz, Alexandra Dent, Shani Buggs, Amanda J. Aubel, Julia Lund, Garen Wintemute, Veronica A. Pear

# Supporting information

**S1 Appendix. Detailed question wording and response options, California Safety and Wellbeing Survey, 2024**

<u>Section I: Gun Violence Restraining Orders</u>

Q1. Have you ever heard of something called a gun violence restraining order (GVRO), extreme risk protection order (ERPO), or “red flag” law?
1. Yes
2. No

*Preamble: California has something called a gun violence restraining order or GVRO law. These are also sometimes called “red flag” laws or extreme risk protection order laws. When someone is threatening to hurt themselves or someone else, and they have or could get a gun, a GVRO can be used to temporarily prevent that person from having or buying guns.*

*The person’s immediate family, roommates, dating partners or individuals with whom they share a child, employers, some co-workers, teachers or school employees, and the police can ask a judge to issue a GVRO. In an emergency, the judge can issue a GVRO immediately that lasts for up to 3 weeks. After a court hearing, the judge can issue a GVRO that lasts for up to 5 years. GVROs are only available if a judge determines that other options to protect against violence have failed or are not appropriate.*

Q2. In general, do you think it would be appropriate for a judge to issue a GVRO in the following scenarios? Assume the person has or could get a gun and other options to protect against violence have failed or are not appropriate.

Statement in row: Randomize
a. The person is experiencing an emotional crisis
b. The person has severe dementia or something like it
c. The person has threatened to physically hurt themselves
d. The person has threatened to physically hurt you or someone else
e. The person has threatened to physically hurt a group of people

Answers in column:
1. Never appropriate
2. Sometimes appropriate
3. Usually appropriate
4. Always appropriate
5. Don’t know

Q3. You mentioned that you didn’t know whether it would be appropriate for a judge to issue a GVRO in one or more scenarios. In a few words or sentences, what additional information would you need to help you decide? [Text Box]

Q4. Would you personally be willing to ask a judge for a GVRO if a member of your family was in one of the following scenarios? Assume your family member has or could get access to a gun and other options to protect against violence have failed or are not appropriate.

Statement in row: Randomize in same order as Q2
a. They were experiencing an emotional crisis
b. They had severe dementia or something like it
c. They had threatened to physically hurt themselves
d. They had threatened to physically hurt you or someone else
e. They had threatened to physically hurt a group of people

Answers in column:
1. Not at all willing
2. Somewhat willing
3. Very willing

Q5. You mentioned that you were not at all willing to ask a judge for a GVRO in one or more scenarios. Please choose the reasons why. Select all that apply.
1. I don’t know enough about GVROs
2. I’m worried about retaliation
3. I’m worried about due-process rights
4. I don’t want to involve the court

5. I don't trust the system to be fair
6. These are personal or family matters
7. It is never appropriate for the government to take a person's guns
8. Other, please specify: [Text Box]

Q6. Would you prefer to have the police ask a judge for a GVRO for you?
1. Yes
2. No

...

Q9. Would you prefer to hold onto your family member's guns yourself, instead of asking a judge for a GVRO?
1. Yes
2. No

Section II: Race and Ethnicity

Are you Spanish, Hispanic, or Latino? Your answer will help represent the entire U.S. population and will be kept confidential. Thank you! Select all answers that apply.
1. No, I am not
2. Yes, Mexican, Mexican-American, Chicano
3. Yes, Puerto Rican
4. Yes, Cuban, Cuban American
8. Yes, other Spanish, Hispanic, or Latino group (Please specify, for example Argentinean, Colombian, Dominican, Nicaraguan, Salvadoran, Spaniard, and so on): [Text box]

Please indicate what you consider your race to be. We appreciate your effort to describe your background using these U.S. Census Bureau categories. Please choose one or more race(s) that you consider yourself to be. Your answer will help represent the entire U.S. population and will be kept confidential. Thank you! Select all answers that apply.
1. White
2. Black or African American
3. American Indian or Alaska Native
4. Asian
5. Native Hawaiian or other Pacific Islander
6. Some other race, please specify: [Text box]

Section III: Firearm Ownership

Q1. Do you or does anyone you live with currently own any type of gun?
1. Yes
2. No
3. Don't know

Q2. Do you personally own a gun?
1. Yes
2. No
