# Supplementary material for: “Has this been tested? Who has it helped? Who has it hurt?”: Public perceptions about California’s extreme risk protection order law
Source: PLoS One. 2025 Nov 4;20(11):e0334967. doi: 10.1371/journal.pone.0334967 (PMC12585041; doi:10.1371/journal.pone.0334967)
Supplement: S1 Table — (PDF) [file pone.0334967.s002.pdf]

“Has this been tested? Who has it helped? Who has it hurt?”: Public perceptions about California’s Extreme Risk Protection Order law

Nicole Kravitz-Wirtz, Alexandra Dent, Shani Buggs, Amanda J. Aubel, Julia Lund, Garen Wintemute, Veronica A. Pear

Supporting information

S1 Table. Sociodemographic Characteristics of Respondents, by Firearm Ownership Status, California Safety and Wellbeing Survey, 2024 (n=3,531)

	Non-owners in homes without guns	Firearm owners	Non-owners who live with firearm owners	Total
Demographic characteristic (population %)	Unweighted *n* (weighted %) [95% CI]	Unweighted *n* (weighted %) [95% CI]	Unweighted *n* (weighted %) [95% CI]	Unweighted *n* (weighted %) [95% CI]
**Age (years)**				
18-29 (21.8)	161 (15.0) [12.6-17.8]	9 (6.5) [3.2-12.5]	28 (20.8) [14.0-29.6]	213 (14.6) [12.6-17.0]
30-44 (27.1)	494 (33.8) [30.8-36.9]	57 (18.9) [14.1-24.9]	77 (29.2) [22.2-37.4]	673 (31.0) [28.6-33.6]
45-59 (24.3)	613 (25) [22.6-27.5]	144 (29.6) [24.5-35.4]	79 (20.4) [14.7-27.7]	891 (25.4) [23.4-27.6]
60+ (26.7)	1,131 (26.2) [24.1-28.5]	408 (45) [39.4-50.7]	149 (29.6) [23.4-36.7]	1754 (28.9) [27.0-30.9]
**Sex**				
Male (49.7)	998 (46.5) [43.5-49.6]	429 (69.4) [63.6-74.5]	75 (27.6) [20.4-36.2]	1588 (48.2) [45.7-50.7]
Female (50.3)	1401 (53.5) [50.4-56.5]	189 (30.6) [25.5-36.4]	258 (72.4) [63.9-79.6]	1943 (51.8) [42.3-54.3]
**Race/ethnicity**				
White (38.4)	1128 (34.9) [32.2-37.6]	406 (58.2) [52.0-64.1]	178 (44.5) [36.6-52.6]	1783 (39.7) [37.3-42.1]
Black (5.5)	154 (5.0) [3.9-6.5]	25 (3.7) [1.9-7.1]	19 (8.0) [4.0-15.1]	215 (5.2) [4.2-6.5]
Latine (36.2)	817 (39.9) [36.9-42.9]	128 (20.5) [16.1-25.6]	100 (28.9) [22.0-36.9]	1118 (35.9) [33.5-38.4]
Asian (15.7)	238 (17.4) [14.9-20.1]	35 (11.2) [7.3-16.8]	24 (11.8) [7.1-19.0]	314 (15.4) [13.5-17.5]
Other/Multi (4.1)	62 (2.9) [0.5-5.9]	24 (6.4) [3.5-11.6]	12 (6.9) [3.4-13.6]	101 (3.9) [2.9-5.0]
**Education**				
Less than high school (14.9)	145 (14.6) [12.3-17.3]	8 (3.2) [1.3-7.9]	9 (5.8) [2.6-12.6]	181 (12.6) [10.7-14.8]
High school (22.1)	321 (22.2) [19.5-25.1]	49 (15.2) [10.8-21.0]	39 (21.9) [15.5-30.0]	450 (21.8) [19.6-24.2]
Some college (30.1)	699 (25.4) [23.0-28.0]	255 (47.6) [41.8-53.5]	108 (38.3) [30.5-46.7]	1112 (30.1) [27.9-32.3]
Bachelor’s or more (32.9)	1234 (37.8) [35.0-10.7]	306 (34.0) [29.0-39.4]	177 (34) [27.2-41.5]	1788 (35.6) [33.3-37.9]
**Household Income**				
<$10,000 (5.8)	168 (5.1) [4.0-6.5]	17 (1.8) [0.9-3.7]	20 (3.3) [1.8-6.0]	227 (4.5) [3.7-5.5]
$10,000-$24,999 (6.9)	261 (5.9) [4.8-7.2]	31 (2.1) [1.2-3.7]	15 (1.2) [0.6-2.5]	328 (4.7) [3.9-5.7]
$25,000-$49,999 (13.6)	454 (15.1) [13.1-17.3]	70 (4.2) [3.1-5.8]	46 (7.3) [7.8-11.0]	606 (12.5) [11.1-14.2]
$50,000-$74,999 (14.3)	331 (13.6) [11.7-15.9]	84 (9.4) [7.1-12.4]	50 (13.5) [8.8-20.1]	498 (13.4) [11.8-15.2]
$75,000-$99,999 (12.6)	337 (12.6) [10.7-14.8]	102 (11.1) [8.2-14.9]	49 (12.1) [8.0-18.1]	515 (12.5) [10.9-14.2]
$100,000-$149,999 (19.0)	375 (18.3) [16.0-20.8]	123 (24.2) [19.4-29.9]	68 (20.0) [14.7-26.7]	587 (19.6) [17.6-21.7]

$150,000+ (27.8)	473 (29.4)<br>[26.6-32.3]	191 (47.1)<br>[41.2-53.1]	85 (42.5)<br>[34.3-51.2]	770 (32.8)<br>[30.4-35.3]
