# Supplementary material for: “Has this been tested? Who has it helped? Who has it hurt?”: Public perceptions about California’s extreme risk protection order law
Source: PLoS One. 2025 Nov 4;20(11):e0334967. doi: 10.1371/journal.pone.0334967 (PMC12585041; doi:10.1371/journal.pone.0334967)
Supplement: S2 Table — (PDF) [file pone.0334967.s003.pdf]

“Has this been tested? Who has it helped? Who has it hurt?”: Public perceptions about California’s Extreme Risk Protection Order law

Nicole Kravitz-Wirtz, Alexandra Dent, Shani Buggs, Amanda J. Aubel, Julia Lund, Garen Wintemute, Veronica A. Pear

Supporting information

S2 Table: Perceived Appropriateness of GVROs, In General, by Risk-Based Scenario, California Safety and Wellbeing Survey, 2024 (n=3,531)

	Never Appropriate		Sometimes Appropriate		Usually/Always Appropriate	
	Unweighted *n*	Weighted % (95% CI)	Unweighted *n*	Weighted % (95% CI)	Unweighted *n*	Weighted % (95% CI)
Person is experiencing an emotional crisis	395	13.8 (12.0-15.8)	1,102	28.3 (26.2-30.6)	1,745	47.0 (44.5-49.5)
Person has severe dementia or something like it	429	13.8 (12-15.7)	646	18.9 (17.0-20.9)	2,157	55.7 (53.2-58.3)
Person threatens to physically hurt themselves	361	11.8 (10.2-13.6)	361	11.8 (10.2-13.6)	2,498	67.5 (65.0-69.9)
Person threatens to physically hurt someone else	318	10.2 (8.7-12.0)	292	8.9 (7.4-10.5)	2,724	73.2 (70.8-75.4)
Person threatens to physically hurt a group of people	332	11.1 (9.5-12.9)	245	7.3 (6.1-8.7)	2,752	73.6 (71.2-75.8)

Note: Percentages may not total to 100% because refusals and don’t know responses are not shown
