# Supplementary material for: “Has this been tested? Who has it helped? Who has it hurt?”: Public perceptions about California’s extreme risk protection order law
Source: PLoS One. 2025 Nov 4;20(11):e0334967. doi: 10.1371/journal.pone.0334967 (PMC12585041; doi:10.1371/journal.pone.0334967)
Supplement: S3 Table — (PDF) [file pone.0334967.s004.pdf]

“Has this been tested? Who has it helped? Who has it hurt?”: Public perceptions about California’s Extreme Risk Protection Order law

Nicole Kravitz-Wirtz, Alexandra Dent, Shani Buggs, Amanda J. Aubel, Julia Lund, Garen Wintemute, Veronica A. Pear

Supporting information

S3 Table: Perceived Appropriateness of GVROs, In General, by Risk-Based Scenario and Firearm Ownership Status, California Safety and Wellbeing Survey, 2024 (n=3,531)

	Never Appropriate		Sometimes Appropriate		Usually/Always Appropriate	
Firearm Ownership Status	Unweighted *n*	Weighted % (95% CI)	Unweighted *n*	Weighted % (95% CI)	Unweighted *n*	Weighted % (95% CI)
Person is experiencing an emotional crisis						
Non-owners in homes without guns	263	13.2 (11.1-15.7)	706	27.3 (24.8-30.0)	1,226	48.4 (45.4-51.5)
Firearm owners	62	12.3 (8.4-17.6)	238	35.9 (30.5-41.7)	283	44.7 (39.0-50.5)
Non-owners who live with firearm owners	36	14.4 (9.3-21.6)	112	26.9 (20.6-34.4)	173	52.7 (44.5-60.8)
Person has severe dementia or something like it						
Non-owners in homes without guns	314	15.1 (12.9-17.6)	402	18.0 (15.7-20.5)	1,471	55.0 (52.0-58.1)
Firearm owners	50	8.7 (5.6-13.4)	152	23.5 (19.1-28.7)	385	61.8 (55.8-67.5)
Non-owners who live with firearm owners	39	13.6 (8.6-20.8)	62	19.9 (14.0-27.5)	216	58.2 (49.7-66.2)
Person threatens to physically hurt themselves						
Non-owners in homes without guns	262	12.5 (6.7-21.9)	248	10.5 (8.8-12.6)	1,721	67.6 (64.6-70.5)
Firearm owners	46	10.1 (6.5-15.6)	113	17.5 (13.3-22.5)	434	68.6 (62.5-74.1)
Non-owners who live with firearm owners	28	11.1 (6.6-18.2)	48	9.1 (5.7-14.2)	250	76.7 (68.9-83.0)
Person threatens to physically hurt someone else						
Non-owners in homes without guns	231	11.0 (9.1-13.1)	166	8.1 (6.5-10.1)	1,857	72.9 (69.9-75.6)
Firearm owners	40	8.8 (5.4-14.	77	10.6 (7.8-14.3)	487	78.6 (73.0-83.3)
Non-owners who live with firearm owners	22	7.4 (4.0-13.2)	27	6.4 (3.6-11.1)	274	80.5 (72.9-86.3)
Person threatens to physically hurt a group of people						
Non-owners in homes without guns	245	12.3 (10.3-14.6)	139	6.3 (4.9-8.0)	1,868	73.1 (70.1-75.8)
Firearm owners	41	8.1 (4.9-13.2)	66	11.8 (8.4-16.2)	495	78.1 (72.4-82.9)
Non-owners who live with firearm owners	19	6.2 (3.2-11.9)	19	3.9 (2.0-7.5)	284	83.1 (75.6-88.6)

Note: Percentages may not total to 100% because refusals and don’t know responses are not shown
