# Supplementary material for: “Has this been tested? Who has it helped? Who has it hurt?”: Public perceptions about California’s extreme risk protection order law
Source: PLoS One. 2025 Nov 4;20(11):e0334967. doi: 10.1371/journal.pone.0334967 (PMC12585041; doi:10.1371/journal.pone.0334967)
Supplement: S4 Table — (PDF) [file pone.0334967.s005.pdf]

“Has this been tested? Who has it helped? Who has it hurt?”: Public perceptions about California’s Extreme Risk Protection Order law

Nicole Kravitz-Wirtz, Alexandra Dent, Shani Buggs, Amanda J. Aubel, Julia Lund, Garen Wintemute, Veronica A. Pear

Supporting information

S4 Table: Perceived Appropriateness of GVROs, In General, by Risk-Based Scenario and Categories of Race and Ethnicity, California Safety and Wellbeing Survey, 2024 (n=3,531)

	Never appropriate		Sometimes appropriate		Usually/Always appropriate	
Race and ethnicity	Unweighted *n*	Weighted % (95% CI)	Unweighted *n*	Weighted % (95% CI)	Unweighted *n*	Weighted % (95% CI)
Person is experiencing an emotional crisis						
White	122	8.7 (6.7-11.2)	682	37.8 (34.4-41.2)	878	46.0 (42.5-49.5)
Black	40	20.7 (12.9-31.4)	48	21.0 (13.1-31.9)	102	46.0 (35.3-57.1)
Latine	187	19.5 (16.0-23.4)	246	20.3 (17.0-24.0)	553	45.2 (40.9-49.5)
Asian	36	12.0 (7.6-18.4)	83	23.5 (18.1-29.9)	171	55.8 (48.4-62.9)
Other/Multi	10	11.6 (4.9-24.9)	43	35.4 (23.5-49.3)	41	40.7 (27.7-55.1)
Person has severe dementia or something like it						
White	127	8.4 (6.6-10.7)	350	20.4 (17.7-23.5)	1,203	63.0 (59.4-66.5)
Black	43	20.0 (12.5-30.3)	41	17.9 (10.8-28.0)	101	43.5 (33.0-54.7)
Latine	203	18.4 (15.2-22.1)	174	17.4 (14.3-21.0)	605	48.5 (44.2-52.9)
Asian	43	15.3 (10.2-22.2)	61	18.6 (13.6-24.9)	187	57.1 (49.7-64.3)
Other/Multi	13	10.6 (4.3-23.8)	20	19.6 (10.3-33.9)	61	58.4 (43.3-72.0)
Person threatened to physically hurt themselves						
White	86	5.8 (4.3-7.8)	258	15.1 (12.6-18.0)	1,365	73.9 (70.5-77.0)
Black	40	24.4 (15.4-36.6)	28	8.8 (5.3-14.3)	127	58.7 (47.3-69.2)
Latine	193	17.4 (14.3-20.9)	109	9.1 (6.8-12.0)	698	59.1 (54.7-63.3)
Asian	34	10.4 (6.5-16.3)	25	8.8 (5.4-14.1)	239	74.5 (67.3-80.6)
Other/Multi	8	9.1 (3.2-23.2)	19	20.6 (11.0-35.3)	69	64.6 (49.3-77.4)
Person threatened to physically hurt someone else						
White	65	4.2 (3.0-5.8)	166	11.1 (8.8-13.8)	1,493	80.2 (76.9-83.1)
Black	38	22.0 (13.3-34.1)	20	7.1 (3.9-12.5)	141	62.6 (51.0-73.0)
Latine	172	15.5 (12.6-18.9)	78	7.4 (5.3-10.2)	764	64.4 (60.1-68.6)
Asian	32	9.3 (5.6-15.2)	20	7.8 (4.6-13.1)	248	77.9 (70.8-83.6)
Other/Multi	11	10.8 (4.4-24.5)	8	5.8 (2.4-13.4)	78	78.0 (63.3-87.9)
Person threatened to physically hurt a group of people						
White	70	4.0 (2.9-5.5)	134	9.1 (7.1-11.6)	1,525	82.6 (79.5-85.3)
Black	42	25.9 (16.4-38.3)	14	3.4 (1.8-6.4)	143	62.1 (50.3-72.7)
Latine	177	17.7 (14.5-21.3)	69	5.8 (4.1-8.1)	761	63.4 (59.0-67.6)
Asian	32	9.1 (5.4-14.8)	18	7.5 (4.3-12.6)	248	77.2 (70.1-83.0)
Other/Multi	11	10.7 (4.2-24.4)	10	7.4 (3.5-15.2)	75	76.2 (61.7-86.4)

Note: Percentages may not total to 100% because refusals and don’t know responses are not shown
