# Supplementary material for: “Has this been tested? Who has it helped? Who has it hurt?”: Public perceptions about California’s extreme risk protection order law
Source: PLoS One. 2025 Nov 4;20(11):e0334967. doi: 10.1371/journal.pone.0334967 (PMC12585041; doi:10.1371/journal.pone.0334967)
Supplement: S5 Table — (PDF) [file pone.0334967.s006.pdf]

"Has this been tested? Who has it helped? Who has it hurt?": Public perceptions about California's Extreme Risk Protection Order law

Nicole Kravitz-Wirtz, Alexandra Dent, Shani Buggs, Amanda J. Aubel, Julia Lund, Garen Wintemute, Veronica A. Pear

Supporting information

S5 Table: Perceived Willingness to Personally Petition for a GVRO for a Family Member, by Risk Scenario, Firearm Ownership Status (Panel A) and Categories of Race and Ethnicity (Panel B), California Safety and Wellbeing Survey, 2024 (n=3,531)

	Not at all willing		Somewhat/very willing	
Firearm ownership status	Unweighted *n*	Weighted % (95% CI)	Unweighted *n*	Weighted % (95% CI)
Person is experiencing an emotional crisis				
Total	650	21.5 (19.4-23.8)	2,843	76.8 (74.4-79.0)
Non-owners in homes without guns	408	19.1 (16.7-21.7)	1,968	79.2 (76.5-81.7)
Firearm owners	127	24.1 (18.9-30.2)	486	75.4 (69.3-80.6)
Non-owners who live with firearm owners	58	22.1 (15.7-30.1)	273	75.6 (67.2-82.3)
Person has severe dementia or something like it				
Total	593	19.9 (17.8-22.1)	2,896	78.2 (75.9-80.4)
Non-owners in homes without guns	394	19.4 (17.0-22.1)	1,976	78.5 (75.8-81.1)
Firearm owners	98	16.5 (12.1-22.0)	516	83.0 (77.5-87.5)
Non-owners who live with firearm owners	51	19.8 (13.5-28.1)	280	77.8 (69.2-84.5)
Person threatened to physically hurt themselves				
Total	403	14.4 (12.5-16.4)	3,095	84.1 (82.0-86.1)
Non-owners in homes without guns	257	13.3 (11.2-15.7)	299	85.4 (82.8-87.6)
Firearm owners	67	13.3 (9.2-18.7)	547	86.3 (80.9-90.3)
Non-owners who live with firearm owners	30	11.9 (7.2-19.0)	299	85.1 (77.3-90.5)
Person threatened to physically hurt someone else				
Total	335	12.0 (10.3-14.0)	3,165	86.4 (84.3-88.2)
Non-owners in homes without guns	219	11.0 (9.1-13.2)	2,161	87.3 (85.0-89.4)
Firearm owners	54	12.1 (8.1-17.7)	561	87.6 (82.0-91.6)
Non-owners who live with firearm owners	19	7.6 (3.9-14.5)	311	89.8 (82.2-94.4)
Person threatened to physically hurt a group of people				
Total	329	11.7 (10.0-13.6)	3,165	86.7 (84.7-88.5)
Non-owners in homes without guns	215	11.2 (9.3-13.4)	2,161	87.3 (84.9-89.3)
Firearm owners	53	11.3 (7.5-16.6)	560	88.2 (82.8-92.0)
Non-owners who live with firearm owners	21	6.5 (3.4-12.1)	310	91.1 (84.3-95.1)

	Not at all willing		Somewhat/very willing	
Categories of race and ethnicity	Unweighted *n*	Weighted % (95% CI)	Unweighted *n*	Weighted % (95% CI)
Person is experiencing an emotional crisis				
Total	650	21.5 (19.4-23.8)	2,843	76.8 (74.4-79.0)
White	288	19.9 (17.0-23.2)	1,482	78.9 (75.5-81.9)
Black	46	23.2 (14.6-34.7)	163	73.1 (61.5-82.2)
Latine	232	23.5 (19.9-27.6)	870	74.3 (70.1-78.1)
Asian	55	17.0 (11.9-23.8)	257	81.2 (74.2-86.7)
Other/Multi	29	34.2 (21.7-49.5)	71	65.6 (50.4-78.2)

Person has severe dementia or something like it				
Total	593	19.9 (17.8-22.1)	2,896	78.2 (75.9-80.4)
White	229	15.6 (12.9-18.6)	1,541	83.4 (80.2-86.1)
Black	55	30.6 (20.7-42.5)	153	65.4 (53.6-75.6)
Latine	232	22.9 (19.4-26.9)	869	74.7 (70.6-78.5)
Asian	53	18.7 (13.1-85.9)	256	78.5 (71.0-84.4)
Other/Multi	24	26.3 (15.1-41.6)	77	73.7 (58.4-84.9)
Person threatened to physically hurt themselves				
Total	403	14.4 (12.5-16.4)	3,095	84.1 (82.0-86.1)
White	138	10.2 (8.0-13.0)	1,633	88.5 (85.6-90.8)
Black	33	13.9 (7.8-23.5)	176	82.4 (72.2-89.4)
Latine	186	18.5 (15.3-22.3)	919	79.8 (75.9-83.2)
Asian	32	15.1 (9.9-22.4)	280	83.5 (75.9-89.0)
Other/Multi	14	15.7 (7.5-29.9)	87	84.3 (70.1-92.5)
Person threatened to physically hurt someone else				
Total	335	12.0 (10.3-14.0)	3,165	86.4 (84.3-88.2)
White	104	7.8 (5.9-10.3)	1,669	91.2 (88.6-93.3)
Black	22	7.4 (4.4-12.2)	187	88.8 (81.8-93.3)
Latine	165	16.2 (13.2-19.8)	941	81.7 (77.9-85.0)
Asian	28	12.3 (7.6-19.1)	284	85.9 (78.7-91.0)
Other/Multi	16	21.7 (11.3-37.6)	84	77.8 (62.0-88.3)
Person threatened to physically hurt a group of people				
Total	329	11.7 (10.0-13.6)	3,165	86.7 (84.7-88.5)
White	99	7.9 (6.0-10.4)	1,670	90.9 (88.2-93.0)
Black	25	7.7 (4.7-12.4)	184	88.6 (81.7-93.1)
Latine	159	15.4 (12.5-19.0)	945	82.8 (79.1-85.9)
Asian	31	12.8 (8.1-19.6)	281	85.4 (78.3-90.5)
Other/Multi	15	17.4 (8.8-31.4)	85	82.4 (68.4-91.0)

Note: Percentages may not total to 100% because refusals and don’t know responses are not shown
