# Supplementary material for: “Has this been tested? Who has it helped? Who has it hurt?”: Public perceptions about California’s extreme risk protection order law
Source: PLoS One. 2025 Nov 4;20(11):e0334967. doi: 10.1371/journal.pone.0334967 (PMC12585041; doi:10.1371/journal.pone.0334967)
Supplement: S6 Table — (PDF) [file pone.0334967.s007.pdf]

“Has this been tested? Who has it helped? Who has it hurt?”: Public perceptions about California’s Extreme Risk Protection Order law

Nicole Kravitz-Wirtz, Alexandra Dent, Shani Buggs, Amanda J. Aubel, Julia Lund, Garen Wintemute, Veronica A. Pear

Supporting information

S6 Table: Perceived Willingness to Personally Petition for a GVRO for a Family Member, by Risk Scenario, Firearm Ownership Status (Panel A) and Categories of Race and Ethnicity (Panel B), California Safety and Wellbeing Survey, 2024 (n=3,531)

Firearm ownership status	Not at all willing		Somewhat willing		Very Willing	
	Unweighted *n*	Weighted % (95% CI)	Unweighted *n*	Weighted % (95% CI)	Unweighted *n*	Weighted % (95% CI)
Person is experiencing an emotional crisis						
Total	650	21.5 (19.4-23.8)	1,558	42.6 (40.1-45.1)	1,285	34.2 (31.9-36.6)
Non-owners in homes without guns	408	19.1 (16.7-21.7)	1,033	29.0 (26.8-31.3)	935	24.1 (22.1-26.2)
Firearm owners	127	24.1 (18.9-30.2)	291	42.0 (36.4-47.8)	195	33.4 (28.2-39.1)
Non-owners who live with firearm owners	58	22.1 (15.7-30.1)	160	44.4 (36.4-52.6)	113	31.2 (24.4-38.9)
Person has severe dementia or something like it						
Total	593	19.9 (17.8-22.1)	1,159	34.0 (31.7-36.5)	1,737	44.2 (41.7-46.7)
Non-owners in homes without guns	394	19.4 (17.0-22.1)	753	33.4 (30.6-36.4)	1,223	45.1 (42.2-48.2)
Firearm owners	98	16.5 (12.1-22.0)	222	38.0 (32.4-43.9)	294	45.1 (39.4-50.9)
Non-owners who live with firearm owners	51	19.8 (13.5-28.1)	120	33.6 (26.4-41.7)	160	44.2 (36.3-52.3)
Person threatened to physically harm themselves						
Total	403	14.4 (12.5-16.4)	993	29.2 (27.0-31.6)	2,102	64.9 (52.3-57.4)
Non-owners in homes without guns	257	13.3 (11.2-15.7)	629	28.7 (25.9-31.5)	1,495	56.7 (53.6-59.7)
Firearm owners	67	13.3 (9.2-18.7)	202	32.1 (26.9-37.8)	345	54.2 (48.2-60.0)
Non-owners who live with firearm owners	30	11.9 (7.2-19.0)	106	27.6 (21.1-35.3)	193	33.6 (23.5-45.5)
Person threatened to physically hurt someone else						
Total	335	12.0 (10.3-14.0)	726	22.6 (20.5-24.8)	2,439	63.8 (61.2-66.2)
Non-owners in homes without guns	219	11.0 (9.1-13.2)	461	24.7 (19.2-24.4)	1,700	65.7 (62.6-68.6)
Firearm owners	54	12.1 (8.1-17.7)	141	23.5 (18.9-28.8)	420	64.1 (58.0-69.8)
Non-owners who live with firearm owners	19	7.6 (3.9-14.5)	76	22.9 (16.7-30.5)	235	66.9 (58.5-74.4)
Person threatened to physically hurt a group of people						
Total	329	11.7 (10.0-13.6)	726	24.3 (22.1-26.7)	2,439	62.4 (59.8-64.9)
Non-owners in homes without guns	215	11.2 (9.3-13.4)	462	24.1 (21.4-27.0)	1,699	63.2 (60.1-66.2)
Firearm owners	53	11.3 (7.5-16.6)	140	23.0 (18.4-28.4)	420	65.1 (59.0-70.7)
Non-owners who live with firearm owners	21	6.5 (3.4-12.1)	73	23.5 (17.1-31.3)	237	67.6 (59.3-75.0)

	Not at all willing		Somewhat willing		Very willing	
Categories of race and ethnicity	Unweighted *n*	Weighted % (95% CI)	Unweighted *n*	Weighted % (95% CI)	Unweighted *n*	Weighted % (95% CI)
Person is experiencing an emotional crisis						
Total	650	21.5 (19.4-23.8)	1,558	42.6 (40.1-45.1)	1,285	34.2 (31.9-36.6)
White	288	19.9 (17.0-23.2)	848	46.0 (42.5-49.5)	634	32.9 (29.8-36.2)
Black	46	23.2 (14.6-34.7)	77	37.9 (27.6-49.5)	86	35.2 (25.8-45.9)
Latine	232	23.5 (19.9-27.6)	158	40.0 (35.8-44.3)	412	34.3 (30.3-38.5)
Asian	55	17.0 (11.9-23.8)	133	43.4 (36.3-50.7)	124	37.8 (31.2-45.0)

Other/Multi	29	34.2 (21.7-49.5)	42	34.3 (22.5-48.3)	29	31.4 (19.7-46.0)
Person has severe dementia or something like it						
Total	593	19.9 (17.8-22.1)	1,159	34.0 (31.7-36.5)	1,737	44.2 (41.7-46.7)
White	229	15.6 (12.9-18.6)	586	34.6 (31.2-38.1)	955	48.8 (45.3-52.3)
Black	55	30.6 (20.7-42.5)	54	29.1 (19.7-40.8)	99	36.3 (27.0-46.8)
Latine	232	22.9 (19.4-26.9)	369	33.0 (29.1-37.2)	500	41.7 (37.5-46.1)
Asian	53	18.7 (13.1-25.9)	113	36.3 (29.5-43.6)	143	42.2 (35.3-49.4)
Other/Multi	24	26.3 (15.1-41.6)	37	35.3 (23.1-49.7)	40	38.4 (25.8-52.9)
Person threatened to physically hurt themselves						
Total	403	14.4 (12.5-16.4)	993	29.2 (27.0-31.6)	2,102	54.9 (52.3-57.4)
White	138	10.2 (8.0-13.0)	505	29.7 (26.5-33.0)	1,128	58.9 (55.3-62.4)
Black	33	13.9 (7.8-23.5)	45	20.8 (13.0-31.7)	131	61.6 (50.2-71.9)
Latine	186	18.5 (15.3-22.3)	311	29.6 (25.8-33.8)	608	50.2 (45.8-54.6)
Asian	32	15.1 (9.9-22.4)	93	28.3 (22.3-35.2)	187	55.2 (47.7-62.4)
Other/Multi	14	15.7 (7.5-29.9)	39	36.6 (24.2-50.9)	48	47.8 (33.9-62.0)
Person threatened to physically hurt someone else						
Total	335	12.0 (10.3-14.0)	726	22.6 (20.5-24.8)	2,439	63.8 (61.2-66.2)
White	104	7.8 (5.9-10.3)	324	20.3 (14.5-23.4)	1,345	71.0 (67.4-74.2)
Black	22	7.4 (4.4-12.2)	47	26.9 (17.3-39.2)	140	61.9 (50.3-72.3)
Latine	165	16.2 (13.2-19.8)	271	25.5 (21.9-29.5)	670	56.2 (51.8-60.5)
Asian	28	12.3 (7.6-19.1)	63	21.0 (15.6-27.8)	221	64.9 (57.2-71.9)
Other/Multi	16	21.7 (11.3-37.6)	21	19.8 (11.1-32.8)	63	58.0 (43.3-71.4)
Person threatened to physically hurt a group of people						
Total	329	11.7 (10.0-13.6)	726	24.3 (22.1-26.7)	2,439	62.4 (59.8-64.9)
White	99	7.9 (6.0-10.4)	325	21.2 (18.2-24.5)	1,345	69.7 (66.1-73.1)
Black	25	7.7 (4.7-12.4)	46	28.5 (18.9-40.4)	138	60.1 (48.7-70.5)
Latine	159	15.4 (12.5-19.0)	270	27.6 (23.8-31.8)	675	55.2 (50.7-59.5)
Asian	31	12.8 (8.1-19.6)	65	24.3 (18.2-31.6)	216	61.1 (53.4-68.4)
Other/Multi	15	17.4 (8.8-31.4)	20	20.6 (11.6-33.9)	65	61.8 (47.3-74.4)

Note: Percentages may not total to 100% because refusals and don’t know responses are not shown
